# Influence of terpenoids and acarbose on glycosyltransferases produced by strain *Leuconostoc mesenteroides* URE 13

**DOI:** 10.1080/13102818.2014.910329

**Published:** 2014-07-08

**Authors:** Veselin Bivolarski, Tonka Vasileva, Petko Bozov, Ilia Iliev

**Affiliations:** ^a^ Department of Biochemistry and Microbiology, Plovdiv University, Plovdiv, Bulgaria

**Keywords:** acarbose, dextransucrase, inhibition, *Leuconostoc mesenteroides*, terpenoids

## Abstract

A study of the influence of different plant terpenoids and amino sugar derivate acarbose on the activity of glycosyltransferase complex and purified dextransucrase from *Leuconostoc mesenteroides* URE 13 strain was carried out. All the tested terpenoids showed an inhibitory effect on glycosyltransferases from strain URE 13 at concentration 0.34 mmol. Out of all studied diterpenoids splendidin showed the strongest inhibitory effect decreasing the activity of both glycosyltransferase complex and dextransucrase with 70% and 90%, respectively. The triterpenoid ursolic showed the second strongest inhibitory effect as the enzyme complex and dextransucrase from strain URE 13 retain 27% and 13% of their initial enzyme activity. Despite the higher degree of inhibition of purified dextransucrase, compared to the enzyme complex, a complete inhibition of the enzyme was not observed at the highest used terpenoid concentration (3.42 mmol). When acarbose was used as an inhibitor, a complete inhibition of dextransucrase was observed at concentration of 6.9 mmol, while the enzyme complex retained 8% of its enzyme activity. Ki values of 0.28 mmol for splendidin, 0.37 mmol for ursolic acid and 0.29 mmol for acarbose were determined from the kinetic studies of purified dextransucrase.

## Introduction

Terpenoids are a large class of natural hydrocarbons produced mainly by plants. Generally, their concentrations are comparatively high in plant reproductive organs, leafs and resins during and immediately after flowering.[1] Up to now, there are more than 40,000 different terpenoid structures found and their number rapidly continue to increase.[2] The enormous variety of terpenoid structures which usually contain oxygen atoms in the form of alcohol, ketone, aldehyde or carboxylic groups is associated with significant differences in their physical and chemical properties, and also with the great number of biological activities displayed by terpenoid compounds.[3] Among the beneficial effects of terpenoids, associated with their application in human healthcare are as follows: prevention and therapy of cancers [4,5]; antimicrobial and antifungal activities [6–8]; anticariogenic activity [9]; antiviral activity [10]; antihyperglycemic activity [11]; antinflamatory activity [12]; and antiparasitic activity.[13] Additionally, terpenoids can found application as skin penetration enhancers during anticancer therapy, and also in dermatology and cosmetology.[1] The anticariogenic effect of terpenoids is associated with their ability to inhibit glucosyltransferases (GTFs) secreted by plaque-forming bacteria belonging to genera *Streptococcus*.[9] Such an inhibitory effect on carbohydrase enzymes, including α-glucosidase, α-amylase, cyclodextrin glucanotransferase and GTFs, is also known for some amino sugar derivatives.[14] One of these derivatives, produced by some strains of *Actinomycetales* sp. is acarbose: pseudotetrasaccharide consisting of two glucose units, 4-amino-4,6-dideoxy glucose unit and unsaturated cyclitol unit. The inhibitory effect of acarbose is ascribed to cyclohexan ring and glycosidic nitrogen linkage that mimics the transition state for cleavage of glycosidic linkages in normal glycosidase substrates.[15,16]

While acarbose is well soluble in aqueous solutions, the terpenoids as lipophilic compounds are soluble only in solutions containing organic solvents and this hampers their study and application. One promising solution for this drawback is the modification of terpenoid molecules by attaching carbohydrate moieties to obtain their glycoside forms.[17] The efficiency of this approach is well demonstrated in plant flavonoids, which useful bioactive properties often can be exploited only in the form of their water soluble glycosyl derivatives. In addition, the glycosides often display different pharmacokinetic properties from these ones of non-glycosylated aglycons, e.g. better solubility, lesser reactivity, different circulation and elimination time, and concentration in body fluids.[3,18] According to that, enzymatic glycosylation of bioactive substances is a perspective technique because of enzyme selectivity and the mildness of reaction conditions, compared to chemical methods, where harsh conditions and toxic catalysts are often used.[19] At this point of view, as useful tools for enzymatic glycosylation of terpenoid compounds appear so-called non-Leloir glycosyltransferases, produced by lactic acid bacteria belonging to genera *Leuconostoc*, *Streptococcus*, *Lactobacillus* and *Weissella*.[20,21] These enzymes are able to transfer a glycosyl residue from a cheap donor such as sucrose to a growing polymer chain, to water (hydrolisis), or to a suitable acceptor (acceptor reaction), using only the energy stored in glycosidic linkage of substrate molecule.[20] Despite the considerable specificity of acceptor reactions which are generally more efficient when carbohydrate acceptors are used, a successful glycosylation of many non-carbohydrate bioactive substances (e.g. aromatic compounds, flavonoids, amino acid derivatives, alcohols) by GTFs from *L. mesenteroides* strains has been achieved.[22,23] According to that, the optimization of the glycosyltransferase reaction, performed with potentially inhibiting and non-carbohydrate acceptor molecules in the presence of water-miscible organic solvents, is a key step in the current enzyme study.

The aim of the present work is to evaluate the influence of different di- and triterpenoids on activity of glycosyltransferase complex and purified dextransucrase produced by *L. mesenteroides* URE 13 strain. We also compared the effect of the studied terpenoids and acarbose on the kinetic of the enzyme reaction catalysed by purified dextransucrase.

## Materials and methods

### Bacterial strains and culture media

*L. mesenteroides* URE 13 was obtained from the bacterial culture collection of the Department of General and Industrial Microbiology, Sofia University (Bulgaria). The strain was cultivated 6–8 h in culture media containing 4% (w/v) sucrose at 27 °C on a rotary shaker (200 rpm) for the production of glycosyltransferases.[24]

### Extraction and isolation of terpenoids

Triterpenoids ursolic acid and oleanolic acid were extracted from dried and finely powdered aerial parts of *Lavandula spica* L. with methanol at room temperature for a week. The methanolic solution was concentrated by evaporation to dryness and residue was chromatographed on silica gel column (Merck, No 7734) as previously described.[25] Diterpenoids scutalpin A, scutalpin E, scutalpin F and scutecyprol A were extracted with acetone from dried and finely powdered stems from species of genera *Scutellaria* (Labiatae), and salviarin, splendidin, splenolide B were extracted from *Salvia splendens*. Diterpenoids were isolated by column chromatography with silica gel as previously described.[26,27,28] Chlorohydrin derivatives of scutalpin A and scutalpin E were prepared by treatment of methanol solution of the compounds with hydrochloric acid.[27] Acetate derivative of ursolic acid was obtained by treatment with acetic anhydride of pyridine solution of the compound, according to Bozov and co-workers.[29] Oxidation of ursolic acid with K_2_Cr_2_O_7_/H_2_SO_4_/acetone solution leads to 3-keto-ursolic acid as described by Bozov and co-workers.[29] All terpenoids were dissolved in dimethyl sulfoxide (DMSO) (Calbiochem).

### Biomass measurements

Bacterial growth was measured by a turbidimetric method at 620 nm and calibrated against cell dry-weight measurements, as previously described.[30]

### Concentration of glycosyltransferases

The culture medium after sucrose cultivation was first centrifuged for 20 min at 7000 g and 4 °C for cell separation. The supernatant was then filtered with a Sartorius membrane (0.2 μm cut-off) to ensure the total absence of cells in the supernatant. The glycosyltransferases were separated from the supernatants and concentrated by using of polyethylene glycol (PEG) 1500 to final concentration of 20% (w/v).[31] The glycosyltransferases were separated by centrifugation at 7000 g for 20 min at 4 °C, collected in the pellet, and diluted in 20 mmol/L sodium acetate buffer, pH 5.3.

### Purification of dextransucrase

An extracellular dextransucrase from *L. mesenteroides* URE 13, cultivated on sucrose media, was purified by size-exclusion chromatography with XK 16/70 column and Sepharose CL-6B medium as previously described.[32]

### Enzyme activity assays

One unit of glycosyltransferase activity is defined as the amount of enzyme that catalyses the formation of 1 μmol of fructose per 1 min at 30 °C in 20 mmol/L sodium acetate buffer (pH 5.3), 0.05 g/L CaCl_2_ and 100 g/L sucrose.[33] Additionally, D-fructose and D-glucose were determined with hexokinase, glucose-6-phosphate dehydrogenase, and phosphoglucose isomerase (commercially available kit, Cat. No. K-FRUGL, Megazyme International Ireland ltd, Wicklow, Ireland). All activities were assayed at least in triplicate and average values are given.

### Determination of inhibitory effect

The inhibitory effect of DMSO on enzyme activity of the glycosyltransferase complex and the purified dextransucrase from strain *L. mesenteroides* URE 13 was studied at concentration from 20% to 70% (v/v) in the reaction mixtures. The inhibitory effect of the studied terpenoids was determined at 0.34, 1.71 and 3.42 mmol per ml end concentration in the reaction mixtures. The inhibitory effect of acarbose (Sigma) was determined at 0.3, 1.3, 2.3, 4.1 and 6.9 mmol per ml end concentration in the reaction mixtures. The reactions were performed with 1 U/ml enzyme in reaction mixtures containing 20 mmol/L sodium acetate buffer (pH 5.3), 0.05 g/L CaCl_2_, 100 g/L sucrose at 30 °C. D-fructose and D-glucose were determined with hexokinase, glucose-6-phosphate dehydrogenase and phosphoglucose isomerase (commercially available kit, Cat. No. K-FRUGL, Megazyme International Ireland ltd, Wicklow, Ireland). All the reactions were performed at least in triplicate and average values are given.

### Determination of Ki values

The Ki values were determined in reaction mixtures containing between 1 and 200 mmol sucrose, terpenoids from 0.34 to 3.42 mmol/ml or acarbose from 0.3 to 6.9 mmol/ml. The reactions were performed with 1 U/ml dextransucrase in 20 mmol/L sodium acetate buffer (pH 5.2) at 30 °C. D-fructose and D-glucose were determined with hexokinase, glucose-6-phosphate dehydrogenase and phosphoglucose isomerase (commercially available kit, Cat. No. K-FRUGL, Megazyme International Ireland ltd, Wicklow, Ireland). All the reactions were performed at least in triplicate and the average values were used for determination of Ki from Lineweaver–Burk plots.

### Protein determination

Proteins were assayed by the method of Bradford [34] by using bovine serum albumin as a standard.

### Statistical analysis

In all the cases the software Programmable scientific calculation ‘CITIZEN’ SRP-45N and SigmaPlot 12.0 (Systat Software, Inc.) were used for data analysis.

## Results and discussion

### Inhibitory effect of DMSO on the activity of glycosyltransferases from *L. mesenteroides* URE 13

**Figure 1.**
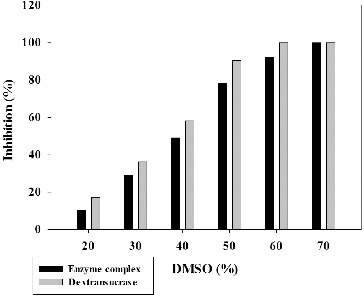
Inhibition of glycosyltransferases from *Leuconostoc mesenteroides* URE 13 by DMSO.

In our previous study, we showed that strain *L. mesenteroides* URE 13 produced a glycosyltransferase complex including 180 kDa dextransucrase, 300 kDa GTF and 120 kDa fructosyltransferase (FTF).[35] The dextransucrase from this complex was purified by single-step chromatography technique.[32] The influence of different concentrations DMSO on the activity of the glycosyltransferase complex and purified dextransucrase was studied. This step is important because the terpenoids have only poor water solubility and usage of compatible organic solvents such as DMSO is required. The lowest inhibitory effect for enzyme complex and dextransucrase was observed in the presence of 20% DMSO – 10.12% and 17.34%, respectively (Figure 1). Increasing the concentration of DMSO leads to increase of the inhibitory effect on the enzyme activity and at the highest used concentration of DMSO (70%) a complete inhibition was reached as for the enzyme complex, thus for dextransucrase (Figure 1). The inhibitory effect on the dextransucrase for all the tested DMSO concentrations was larger than this one of the enzyme complex, and a complete inhibition of the purified enzyme was observed at 60% DMSO (Figure 1). On the basis of these results, we performed the next inhibitory effect studies using terpenoid compounds dissolved in DMSO with 20% final concentration in the reaction mixtures.

In addition to their ability to synthesize polymers, glycosyltransferases produced by lactic acid bacteria also catalyse the transfer of glucose or fructose units from sucrose to other carbohydrate acceptors in so-called acceptor reactions.[36] Of great interest is the potential application of these enzymes for acceptor reactions using non-conventional acceptor molecules that are usually not sugars. Most of them (alkylglycosides, arylglycosides, flavonoids, terpenoids) have poor water solubility, and because of that the use of organic solvents, such DMSO could allow them to dissolve and make their interaction with the enzyme more effective. In the present study we have found that at 20% concentration of DMSO, which ensures good solubility of the studied terpenoids, glycosyltransferase complex and the purified dextransucrase from strain URE 13 retain about 90% and 83% of their enzyme activity. In other study, Girard and Legoy found that dextransucrase from *L. mesenteroides* NRRL B-512F loses half of its enzyme activity in the presence of 20% DMSO.[37] It appears that the different degree of inhibition of both NRRL B-512F dextransucrase and the enzyme from URE 13 is a strain dependent feature. Girard and Legoy proposed two hypotheses for the inhibitory effect of DMSO: (1) the increase of DMSO concentration to 50% leads to a change of pH from 5.0 to 6.0 which is above the reported optimum for the action of these enzymes and (2) the presence of water soluble organic solvent could modify the nature and the number of non-covalent interactions (Van der Waals, electrostatic, hydrophobic or hydrogen bonds).[37]

### Study of inhibitory effect of terpenoids on the activity of glycosyltransferases from *L. mesenteroides* URE 13

**Figure 2.**
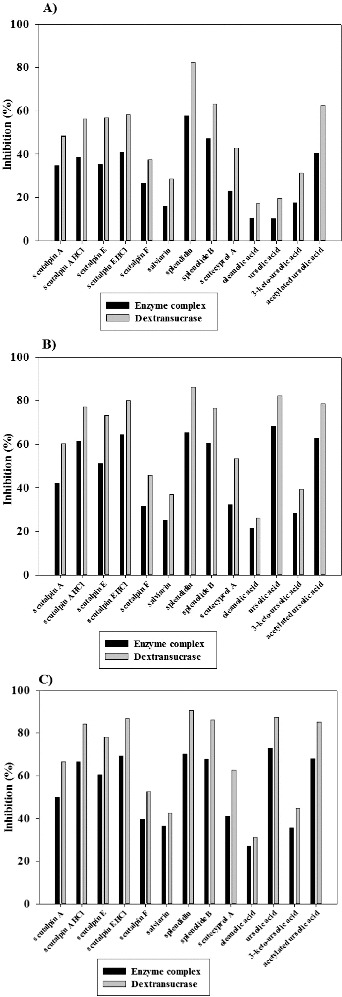
Inhibition of glycosyltransferases from *Leuconostoc mesenteroides* URE 13 by terpenoids at concentrations 0.34 mmol (A), 1.71 mmol (B) and 3.42 mmol (C).

In order to determine the inhibitory effect of terpenoides on the activity of glycosyltransferase complex, as well as on the purified dextransucrase from *L. mesenteroides* URE 13, we tested seven diterpenoids, two triterpenoids and also four their derivatives in three different concentrations (Figure 2). In all the cases the inhibition of dextransucrase was about 10%–25% larger than this of glycosyltransferase complex. From the studied diterpenoids, the highest inhibitory effect was detected in the presence of splendidin in all the tested concentrations. This tendency was observed as for the enzyme complex, thus for purified dextransucrase (Figure 2). Even at the lowest used concentration of splendidin (0.34 mmol), 57% and 82% inhibition of the enzyme complex and dextransucrase were detected (Figure 2(A)). The second highest inhibitory effect was detected in the presence of splenolide B – 47% for the enzyme complex and 63% for dextransucrase (Figure 2(A)). The further increase of inhibitory concentrations did not lead to linear increase of the degree of inhibition. A complete inhibition of the enzyme complex as well as of the dextransucrase was not observed at the highest used concentration of splendidin (3.42 mmol) at which about 30% and 10% of the activity was retained (Figure 2(C)). From both studied diterpenoid derivatives, scutalpin E HCl showed higher degree of inhibition to glycosyltransferase complex and the purified dextransucrase – 69% and 87%, respectively (Figure 2(C)). As scutalpin A thus scutalpin E derivatives showed 9%–18% higher degree of inhibition compared to native substances (Figure 2).

The analysis of the effect of the triterpenoids ursolic acid and oleanolic acid showed that the degree of inhibition increased with their concentration. The highest percent of inhibition was reached when the ursolic acid was applied in concentration 3.42 mmol – 73% for the enzyme complex and 87% for the dextransucrase (Figure 2(C)). Studied derivatives of ursolic acid showed higher inhibitory effect only at the lowest concentration (0.34 mmol) (Figure 2(A)). At final concentrations of ursolic acid set at 1.71 and 3.42 mmol in the reaction mixtures, its inhibitory effect increased and was larger than this of ketone and acetylated derivatives (Figures 2(B) and  2(C)). Similar to the studied diterpenoids, ursolic acid and its derivatives did not show a complete inhibition of the glycosyltransferases from *L. mesenteroides* URE 13 at the highest used concentrations in the assays.

In this paper, we describe for the first time the influence of terpenoids on the activity of dextransucrase produced by *L. mesenteroides* URE 13. Lower degree of inhibition of the enzyme complex compared to dextransucrase could be explained with the presence of two types of glycosyltransferases (GTF and FTF) in the complex. The lack of a complete inhibition at the highest used concentration of terpenoids (3.42 mmol) gives us an opportunity for further detail studies concerning application of glycosyltransferases as a useful tool for glycosylation of natural substances with low water solubility. Several studies have reported successful glycosylation of non-carbohydrate substances as aromatic compounds, alcohols and amino acid derivatives in which the transferred sugar moieties improves not only their water solubility but also their pharmacological properties.[22] Terpenoid concentrations larger than 3.42 mM were not tested because of their low solubility in the reaction mixtures, containing 20% DMSO.

### Study of inhibitory effect of acarbose on the activity of glycosyltransferases from *L. mesenteroides* URE 13

**Figure 3.**
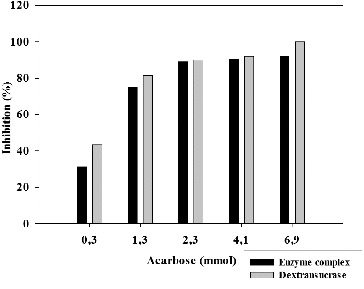
Inhibition of glycosyltransferases from *Leuconostoc mesenteroides* URE 13 by acarbose.

The inhibitory effect of the amino sugar derivative acarbose on the enzyme activity of glycosyltransferase complex and the purified dextransucrase from *L. mesenteroides* URE 13 was studied. At all the tested acarbose concentration the inhibitory effect on dextransucrase activity was predominant (Figure 3). More than 30% and 40% of inhibition was observed for the enzyme complex and the dextransucrase at the lowest tested concentration of acarbose – 0.3 mmol. When the concentration of acarbose was raised to 6.9 mmol, a complete inhibition of the dextransucrase was achieved, while the glycosyltransferase complex retained 8% of its enzyme activity (Figure 3). Similar to the inhibition by terpenoids, at acarbose concentrations larger than 0.3 mmol the dose/effect relation loses its linear dependence.

### Kinetic study of the influence of splendidin, ursolic acid and acarbose on dextransucrase from *L. mesenteroides* URE 13

**Figure 4.**
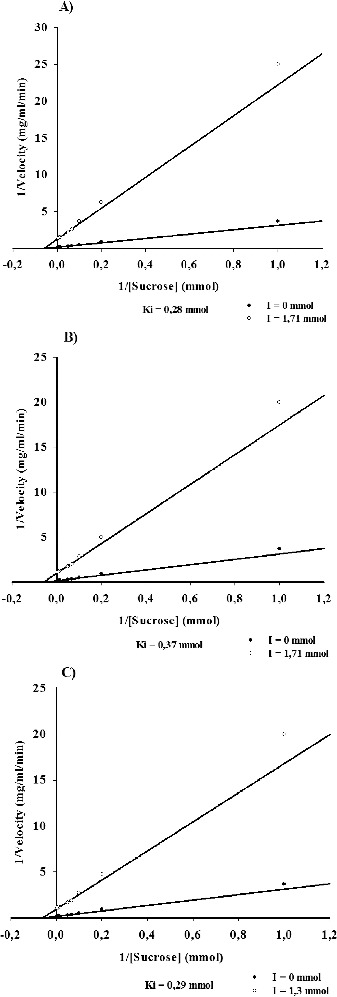
Ki values of dextransucrase inhibition by splendidin (A), ursolic acid (B) and acarbose (C).

Km value of the studied enzyme was determined at 18.0 mmol sucrose, and also an effect of substrate inhibition is observed at sucrose concentrations larger than 200 mmol (data not shown). In order to study the influence of the terpenoids splenididin and ursolic acid, and also of the amino sugar acarbose on velocity of the reaction catalysed by dextransucrase from strain URE 13, we determined and compared their inhibitory constants (Figure 4). In the presence of splenididin or acarbose, very similar values of Ki were determined – 0.28 mmol and 0.29 mmol, respectively (Figures 4(A) and  4(C)). When ursolic acid was applied to the reaction mixture a Ki value of 0.37 mmol was obtained (Figure 4(B)). Based on these results, it could be concluded that splenididin and ursolic acid are at least as effective inhibitors of dextransucrase from strain URE 13 as acarbose.

The amino sugar acarbose is known as a potent inhibitor of several carbohydrases, including α-amylase, cyclodextrin glucanotransferase, α-glucosidase and GTFs.[14,38] As an inhibitor, acarbose is used for treatment of non-insulin-dependent diabetes,[39] and also represent an interest for potential application as an anticaries agent, inhibiting the formation of insoluble glucan by glucansucrases from cariogenic strains of *Streptococcus* spp.[16] Kim and co-workers have found that the addition of acarbose inhibits the formation of dextran by dextransucrase from *L. mesenteroides* B-512FMCM. The authors also defined acarbose as a non-competitive inhibitor with a Ki of 1.35 mmol.[15] For dextransucrase produced by *L. mesenteroides* URE 13, we obtained Ki of 0.29 mmol when acarbose was used as an inhibitor. Similar value of Ki was also obtained for the diterpenoid splendidin – 0.28 mmol. The ursolic acid was also determined as an effective inhibitor of the studied dextransucrase with Ki of 0.37 mmol. Kim and co-workers have reported also that acarbose influences the amount and type of the products obtained during the acceptor reactions catalysed by dextransucrase.[15] In relation to that, the obtained results are a good base for further studies of the acceptor reaction catalysed by dextransucrase from *L. mesenteroides* URE 13 in the presence of terpenoids and acarbose as well. On the other hand, studied terpenoid compounds show significant inhibitory capabilities, which represent a potential interest for their application as anticaries agents. Such information is already available for triterpenoids ursolic acid and oleanolic acid.[9]

## Conclusions

Glycosyltransferase complex and dextransucrase produced by *L. mesenteroides* URE 13 showed a different degree of inhibition in the presence of terpenoids and acarbose as the purified enzyme was more sensitive. From the studied diterpenoids, the highest inhibitory effect was detected in the presence of splendidin – 90% inhibition of dextransucrase, and the triterpenoid ursolic acid showed 87% inhibition of the enzyme. The derivatives of scutalpin A and scutalpin E showed 9%–18% higher inhibitory effect than the native diterpenoids, while the ketone and the acetylated derivatives of ursolic acid caused 10%–40% higher inhibition of dextransucrase only at the lowest tested concentrations – 0.34 mmol. At the highest used concentration, none of the tested terpenoids did not show a complete inhibition of the enzyme complex and dextransucrase. When the inhibitory effect of acarbose was tested, a complete inhibition of dextransucrase was achieved at concentration 6.9 mmol, while the enzyme complex retained 8% of its enzyme activity. As for terpenoids thus for acarbose the dose/effect relation loses its linear dependence at concentrations larger than 0.3 mmol. The determination of inhibitory constant of dextransucrase from strain URE 13 when splendidin and ursolic acid were used as inhibitors showed that both terpenoids have very near Ki values – 0.28 and 0.37 mmol, to this one of acarbose – 0.29 mmol. The obtained results give us a good base for further investigations directed to study the influence of terpenoids on acceptor reactions catalysed by dextransucrase from strain URE 13 and also their glycosylation which will improve water solubility and probably biological activity of these compounds. 
